# Team idiosyncratic deals and team breakthrough innovation: Based on the perspective of input-process-output model

**DOI:** 10.3389/fpsyg.2022.974569

**Published:** 2022-09-06

**Authors:** Zili Fan, Hao Sun, Lijun Wang, Mengting Zhu, Ting Peng

**Affiliations:** ^1^School of Economics and Management, Hubei University of Technology, Wuhan, China; ^2^School of Management, Shanghai University, Shanghai, China

**Keywords:** team idiosyncratic deals, team transactive memory systems, team cognitive flexibility, team exploratory-exploitative knowledge sharing, team breakthrough innovation

## Abstract

**Purpose:**

As a new human resource management practice, idiosyncratic deals are personalized employment arrangements negotiated between employees and employers and intended to benefit them both. It plays an important role in attracting, retaining and motivating employees to promote breakthrough innovation. Based on the input-process-output (I-P-O) model, this paper examines the relationship between team idiosyncratic deals and team breakthrough innovation, the mediating role of team exploratory-exploitative knowledge sharing, and the moderating roles of team transactive memory systems and team cognitive flexibility.

**Participants and methods:**

In order to reduce the effects of common method biases and causal lag effect, this study is divided into three stages for data collection, with a time interval of 1 month. Eighty teams (406 employees) from six enterprises in Shanghai and Hangzhou were selected as samples, and the hypothesis test was carried out by hierarchical regression analysis, bootstrap, and Johnson-Neyman method.

**Results:**

The results show that higher team idiosyncratic deals are associated with higher team breakthrough innovation through higher team exploratory-exploitative knowledge sharing, and that team transactive memory systems and team cognitive flexibility positively moderate the mediating effect of team exploratory-exploitative knowledge sharing in the relationship between team idiosyncratic deals and team breakthrough innovation in the first stage and the second stage, respectively. Under the joint effect of high team transactive memory systems and high team cognitive flexibility, the mediating effect of team exploratory-exploitative knowledge sharing is stronger.

**Conclusion:**

The research results break through the previous research framework of social exchange theory, and I-P-O model to explore the influence mechanism of team idiosyncratic deals, in order to promote the sustainable growth of team breakthrough innovation through this non-standard work arrangement. It is hoped that this research can inspire modern enterprises to create team idiosyncratic deals for valuable teams engaged in breakthrough innovation, which are more conducive to give full play to their heterogeneous talents, and finally help enterprises break through the industry bottleneck and win the market competition.

## Introduction

With the flat development of organizational structure, teams have gradually become the basic work units for enterprises to cope with competitive challenges (Jia et al., 2014). Especially in the breakthrough innovation activities, more and more enterprises regard the team as the main force for breakthrough innovation, because the team resource level is an important driving force. Among them, idiosyncratic deals (I-deals) owned by the team are considered as important resources, because I-deals not only meet the external needs of teams, but also meet the internal needs (Hornung et al., 2010).

Team I-deals are voluntary, personalized agreements of a non-standard nature negotiated between valuable teams and their employers regarding terms that benefit each party (Anand et al., 2010), which include developmental I-deals, flexible I-deals, task I-deals, *ex ante* I-deals, *ex post* I-deals, etc. Team I-deals have characteristics of high cost, so they can only be used for high-value teams in the organization. The organization also places high hopes on these valuable teams, hoping to improve their breakthrough innovation through meeting their unique needs, and finally help the organization achieve new breakthroughs. Therefore, this paper chooses breakthrough innovation as the consequence of team I-deals.

Breakthrough innovation is a process, product or service innovation that has unprecedented performance characteristics, or can significantly improve performance to changing existing markets or creating new markets. It means a new field, it is a process of contradiction between new and old ideas, goals and competitive needs (Shao et al., 2019). In this process, the team not only needs to understand and expand existing knowledge, but also needs to study and develop new knowledge. Therefore, a pair of mutually competitive knowledge sharing behaviors at the innovation level have been formed, that is, exploitative knowledge sharing and exploratory knowledge sharing. Meanwhile, Team I-deals not only allow teams to enjoy more organizational resources, but also make teams to undertake more complex tasks. Thus, teams need to strengthen the knowledge sharing (Anand et al., 2021). Given all of that, the first purpose of this paper is choosing knowledge sharing as the mediating variable to explore how team I-deals (input) are related to team breakthrough innovation (output) through team exploratory-exploitative knowledge sharing (process) based on I-P-O (input-process-output) model.

In the process of team I-deals influencing the generation of team breakthrough innovation, the external environment and the cognitive level will affect the teams’ interpretation of I-deals. Firstly, in terms of the external environment, previous studies have shown that team transactive memory systems (TTMS) have a significant positive impact on both exploratory knowledge sharing and exploitative knowledge sharing (Lewis, 2003). This paper speculates that compared with a team under low level TTMS, the team under a high TTMS is more likely to respond to the team I-deals by means of knowledge sharing. Secondly, in terms of team cognitive level, how to dialectically view the relationship between exploitation and exploration in the actual situation, then promote the generation of breakthrough innovation, the team cognitive flexibility (TCF) is particularly important (Zhu, 2008). Accordingly, the second purpose of this paper is to explore how TTMS and TCF moderate the mediating effect of team exploratory-exploitative knowledge sharing in the relationship between team I-deals and team breakthrough innovation.

**FIGURE 1:**
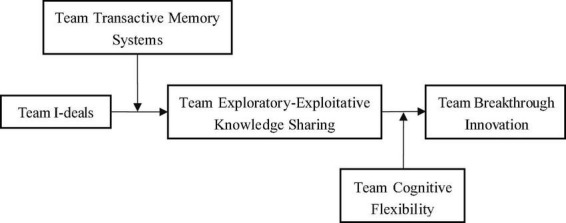
Theoretical model.

To sum up, this paper establishes a two-stage moderated mediating model, as shown in Figure 1. Firstly, based on the I-P-O model, this paper reveals the mediating role of team exploratory-exploitative knowledge sharing in the relationship between team I-deals and team breakthrough innovation. Secondly, TTMS and TCF are introduced to analyze their moderating roles in the process.

## Literature review and hypothesis deduction

### Idiosyncratic deals

For a long time, human resource management has emphasized consistency and standardization (Greenberg et al., 2004). However, in recent years, with the deepening of employees’ self-awareness, the traditional standardized management practice has been unable to meet the needs of employees’ uniqueness (Lai et al., 2009). Those employees who seek to learn knowledge, skills, work value and significance at work begin to negotiate with the organization about their own demands. The emergence of I-deals challenges various theoretical assumptions in the past human resource management (Marescaux et al., 2017), and provides a new way for organizations to effectively motivate employees. I-deals emphasizes the non-standard work arrangement of equal negotiation and voluntary signing between employers and employees (Rousseau, 2006).

Previous studies have found that the flexible work arrangement created by I-deals can meet the unique needs of valuable employees (Hornung et al., 2008), convey organizational care and recognition signals (Anand et al., 2010), and help to enhance the intrinsic motivation of valuable employees (Liu et al., 2013; Wang et al., 2019), organizational self-esteem (Thakur, 2020) and innovative self-efficacy (Liao et al., 2016), and then promote innovation engagement (Hornung et al., 2010) and innovation performance (Wang et al., 2018).

However, previous studies mostly discussed the impact of I-deals on individual employees, and lacked consideration of other subjects. In the meta-analysis of I-deals, Liao et al. (2016) pointed out that as an incentive means, I-deals are impossible to operate in a vacuum, and its implementation must be affected by multiple subjects. Among them, as the main operating unit in modern organizations, team plays an irreplaceable role in the implementation of I-deals (Liao et al., 2016). Therefore, in future research, I-deals should be raised to the team level to more comprehensively explore the impact mechanism of I-deals.

### The mediating role of team exploratory-exploitative knowledge sharing

Firstly, team I-deals will generate a higher level of team vitality (Oh and Kang, 2018; Huang and Chen, 2021). Driven by the high team vitality, the communication and coordination among members have been strengthened, and team members are more willing to share the knowledge they have learned. Secondly, team I-deals enhances team efficacy through the cooperation among team members, which is the most powerful predictor of knowledge sharing. Finally, higher team I-deals represent a supportive work environment full of mutual respect and trust (Anand et al., 2021). Trust and respect are important prerequisites for sharing behavior (Haesebrouck et al., 2021), In order to achieve the expected team results, team members will be more inclined to strengthen the overall competence of the team through knowledge sharing.

At the same time, in the face of the requirements of breakthrough innovation, team not only need existing knowledge as the foundation (exploitative knowledge sharing), but also need brand-new knowledge to promote breakthrough (exploratory knowledge sharing). Therefore, in the process of knowledge sharing, both exploratory knowledge sharing and exploitative knowledge sharing are indispensable. Accordingly, we draw the following assumptions:

**Hypothesis 1:** Team I-deals are positively associated with team exploratory-exploitative knowledge sharing.

Furthermore, team breakthrough innovation needs a large amount of team knowledge reserves as support. Knowledge sharing increases the knowledge resources available to the team, provides important support for breakthrough innovation. Accordingly, in combination with hypothesis 1, we propose the following assumptions:

**Hypothesis 2:** Team exploratory-exploitative knowledge sharing mediates the relationship between team I-deals and team breakthrough innovation.

### The moderating role of team transactive memory systems

According to social information processing theory, individuals will adjust their attitude and behavior toward work according to environmental information (Pfeffer, 1978). As a kind of important environmental information, the TTMS reflects the dependence and trust of team members on each other’s professional knowledge (Peltokorpi, 2008). When TTMS is high, team members are more inclined to establish a close communication network with others, effectively and accurately capture the type of knowledge they need (Austin, 2003). Therefore, if this type of team is motivated by team I-deals, team members will actively communicate with others and share their reserved knowledge in the team to improve the probability of achieving team goals. When the TTMS is low, communication in the team is inefficient, which will cause the waste and idleness of team resources. Signing team I-deals with them makes it difficult for the team to respond to team I-deals through knowledge sharing due to poor coordinated communication. Accordingly, we propose the following assumptions:

**Hypothesis 3:** TTMS moderate the relationship between team I-deals and team exploratory-exploitative knowledge sharing. That is, the higher TTMS, the stronger team I-deals are associated with team exploratory-exploitative knowledge sharing.

Further, with the diffusion of knowledge sharing, the amount of knowledge available to team members is greater than their own reserves, which makes the team work efficiency achieve the effect of 1 + 1 > 2. With the improvement of the knowledge sharing, it is more conducive to the enhancement of breakthrough innovation of the team. Therefore, we propose the following assumptions in combination with hypothesis 3:

**Hypothesis 4:** TTMS moderate the mediating effect of team exploratory-exploitative knowledge sharing. That is, the higher TTMS, the stronger the mediating effect of team exploratory-exploitative knowledge sharing in the relationship between team I-deals and team breakthrough innovation.

### The moderating role of team cognitive flexibility

Knowledge sharing has loaded the team with sufficient knowledge reserves. However, whether the team can achieve the goal, it still needs to select the appropriate knowledge content in the specific external context to avoid inefficient output. In response to this problem, this study believes that TCF plays a key role in this process. According to previous studies, TCF is considered to be a team cognitive ability that can flexibly construct and transform cognitive structure, which is specifically reflected in the flexible transformation of psychological representations by team members in different situations, adjust the information processing mode, and find solutions to different problems (Müller et al., 2015). With a high level of TCF, team members can quickly make multidimensional and in-depth interpretation of these knowledge and their relevance, and give more different knowledge combinations through extensive association (Dina and Da, 2015), so as to more effectively promote the generation of team breakthrough innovation. On the contrary, under the low level of TCF, team members tend to interpret the acquired knowledge in a fixed mode of thinking, which is very easy to cause the deviation between knowledge, which is not conducive to the initiation of new ideas and thus inhibits the breakthrough innovation of the team. Accordingly, we propose the following assumptions:

**Hypothesis 5:** TCF moderates the relationship between team exploratory-exploitative knowledge sharing and team breakthrough innovation, that is, the higher TCF, the stronger team exploratory-exploitative knowledge sharing is associated with team breakthrough innovation.

Furthermore, when a team with high TCF is faced with team I-deals, it is more conducive for it to recognize the expectations that the organization conveys to the team through I-deals, and make full use of the opportunity of team knowledge sharing, select the knowledge structure that is conducive to the generation of innovation, and promote the generation of team breakthrough innovation (Vries et al., 2015). However, in the face of team I-deals, a team with low TCF may not fully realize that this non-standard work arrangement is both an opportunity and a challenge. Then, it is more inclined to avoid risks than to take advantages of knowledge sharing to strive for the upper reaches (Steenbergen et al., 2015), which ultimately reduces the promotion of team breakthrough innovation. Accordingly, in combination with Hypothesis 5, we propose the following assumptions:

**Hypothesis 6:** TCF moderates the mediating effect of team exploratory-exploitative knowledge sharing, that is, the higher TCF, the stronger mediating effect of team exploratory-exploitative knowledge sharing in the relationship between team I-deals and team breakthrough innovation.

### Joint effect of team transactive memory systems and team cognitive flexibility

Integrating the above assumptions, I-deals are signed with the high TTMS team. While team members enjoy more work resources, the organization also has higher work requirements for them (such as breakthrough innovation), which is conducive to stimulating the vitality of team members, promoting team interaction, and responding to team I-deals through team knowledge sharing. However, in this process, because the generation of team breakthrough innovation requires both the existing exploitative knowledge and the new exploratory knowledge, it promotes the formation of exploratory-exploitative knowledge sharing. Subsequently, the high TCF will further stimulate the team to flexibly screen and output the two types of shared knowledge, seek advantages and avoid disadvantages by using exploratory-exploitative knowledge sharing to more specifically strengthen the increase of team knowledge reserve, and then promote the generation of team breakthrough innovation. Accordingly, this study proposes a two-stage moderated mediating model, and further puts forward the following assumptions:

**Hypothesis 7:** TTMS and TCF jointly moderate the mediating effect of team exploratory-exploitative knowledge sharing. That is, the higher TTMS and TCF, the stronger mediating effect of team exploratory-exploitative knowledge sharing in the relationship between team I-deals and team breakthrough innovation.

## Participants and methods

### Participants and procedures

This study conducted field visits to six high-tech enterprises in Hangzhou and Wuhan. We have used cross-sectional design. All the surveys were carried out in three stages from March to May 2022, with an interval of 1 month among each stage. In order to facilitate the orderly development of the survey, in each stage of the survey, the teams entered a meeting room by turn to fill in the questionnaire. The subjects kept a certain space distance and no direct leaders were present. Before filling in the questionnaire, the researcher clearly informed the subjects that the results of the questionnaire were only used for academic research, the answer results of the subjects shall be kept strictly confidential, and there is no right or wrong answer in the questionnaire. Thus the respondents shall fill in the form according to their actual situation. After the subjects completed the questionnaires, the researchers collected the questionnaires on the spot, and screened the questionnaires after the subjects left the meeting room to eliminate the questionnaires with regular errors.

In the first stage, we collected team I-deals, TTMS, and demographic variables. After a month, in the second stage, we collected team exploratory-exploitative knowledge sharing, TCF. After another month, in the third stage, we collected team breakthrough innovation. The entire process questionnaire was strictly coded. A total of 432 employees participated in the questionnaire. After excluding invalid questionnaires, a total of 406 employee questionnaires were obtained from 80 teams, with a team size of 3–12 people.

Among the participants, men accounted for 59.36% and women accounted for 40.64%. In terms of education, only 3.20% have high school education or below, 7.88% have junior college degrees, 73.40 and 14.78% have undergraduate and postgraduate degrees, and 0.74% have doctoral degrees. In terms of age distribution, 9.11% of employees are 18–25 years old, 73.15% are 26–35 years old, 9.85% are 36–45 years old, 7.14% are 46–55 years old, and only 0.75% are over 56 years old%. In terms of working years, the number of employees with less than 1 year accounted for 2.46%, the number of employees with 1–2 years accounted for 7.64%, the employees with 3–5 years accounted for 26.60% of the total sample, and the employees with 6–10 years accounted for 49.26%, 14.04% of the employees have worked for 10 years or more.

### Measures

The scale is from mainstream literature and has been verified for local applicability. And then, we use back translation to translate the English scale. In addition to the control variables, the variable measurement adopts the 5-point Likert scale, ranging from 1 (extremely disagree) to 5 (extremely agree). In order to ensure the applicability of the questionnaire, we selected a small number of local enterprises for the applicability test before the questionnaire survey.

#### Team I-deals

Using the scale of Hornung et al. (2014), with a total of nine items. A sample item is: “Extra flexibility in starting and ending my work day.” Cronbach’s alpha was 0.944.

#### Team exploratory-exploitative knowledge sharing

The 6-item scale developed by Han Ying and Chen Guohong is used. A sample item is: “Team members are willing to share their useful experience with you.” Cronbach’s alpha was 0.890.

#### Team breakthrough innovation

Using the 4-item scale developed by Menguc et al. (2014), a sample item is: “Innovations that fundamentally change existing products.” Cronbach’s alpha was 0.922.

#### Team transactive memory systems

The 15-item scale developed by Lewis (2003) is used. A sample item is: “Different team members are responsible for expertise in different areas.” Cronbach’s alpha was 0.932.

#### Team cognitive flexibility

Using the 12-item scale developed by Martin and Rubin (1995), A sample item is: “The team can adapt to the unfamiliar environment.” Cronbach’s alpha was 0.935.

## Results

### Descriptive statistics

Descriptive statistics and correlation coefficients between variables were shown in Table 1. Team I-deals was positively associated with exploratory-exploitative knowledge sharing (*r* = 0.579, *p* < 0.001), and exploratory-exploitative knowledge sharing was positively associated with team breakthrough innovation (*r* = 0.562, *p* < 0.001).

**TABLE 1:** Means, standard deviations, and correlation matrix for key measures.

Variables	*Mean*	*SD*	1	2	3	4	5	6	7	8	9
1. Team average gender	0.406	0.236	–								
2. Team average age	2.153	0.345	0.123	–							
3. Team average education	3.036	0.352	0.258*	–0.051	–						
4. Team average tenure	3.633	0.511	0.136	0.742***	–0.031	–					
5. Team size	5.090	1.778	–0.002	0.167	–0.130	0.078	–				
6. Team I-deals	3.536	0.847	–0.097	0.048	0.065	0.307**	0.137	–			
7. Team exploratory-exploitative knowledge sharing	2.484	0.800	0.040	0.059	–0.101	0.188	0.261*	0.579***	–		
8. Team breakthrough innovation	2.793	0.669	0.040	0.029	0.023	0.258*	0.239*	0.539**	0.562***	–	
9. TTMS	4.005	0.495	–0.015	0.081	0.102	0.153	0.017	0.440***	0.230*	0.129	–
10. TCF	3.813	0.542	–0.080	0.029	0.215	0.003	–0.149	0.081	−0.258*	0.068	0.385***

*N* = 80. SD, Standard deviation. *p < 0.05, **p < 0.01, ***p < 0.001. This table shows the correlation, mean, and standard deviation of the variables at the team level. And “team”-values are calculated by averaging over the team members.

### Hypothesis testing

**Firstly, we test hypothesis 1**. The result of model 5 in Table 2 shows that team I-deals are positively related to team exploratory-exploitative knowledge sharing (β = 0.557, *p* < 0.001). So hypothesis 1 was supported.

**TABLE 2:** Hierarchical regression results.

Variables	Team breakthrough innovation								Team exploratory-exploitative knowledge sharing					
	Model 1		Model 2		Model 3		Model 4		Model 5		Model 6		Model 7	
	β	*s.e.*	β	*s.e.*	β	*s.e.*	β	*s.e.*	β	*s.e.*	β	*s.e.*	β	*s.e.*
Intercept	2.056*	0.796	1.856*	0.757	2.012**	0.746	2.413**	0.692	3.161**	0.917	3.154**	0.925	3.455***	0.871
Team average gender	0.220	0.284	0.078	0.273	0.121	0.268	0.063	0.246	0.482	0.327	0.483	0.329	0.326	0.312
Team average age	–0.450	0.290	–0.451	0.275	–0.510	0.271	−0.526*	0.248	0.002	0.334	0.006	0.337	0.095	0.317
Team average education	–0.008	0.189	0.096	0.182	0.035	0.181	–0.017	0.167	–0.354	0.218	–0.352	0.220	–0.382	0.206
Team average tenure	0.349	0.205	0.363	0.194	0.393*	0.191	0.376*	0.175	–0.048	0.236	–0.050	0.238	–0.166	0.225
Team size	0.073	0.037	0.052	0.036	0.058	0.035	0.046	0.032	0.073	0.043	0.073	0.043	0.072	0.040
Team I-deals	0.355***	0.084	0.191	0.096	0.140	0.098	0.105	0.090	0.557***	0.097	0.563***	0.107	0.512***	0.102
Team exploratory- exploitative knowledge sharing			0.294**	0.096	0.358**	0.100	0.377***	0.091						
TTMS											–0.021	0.165	0.379	0.196
Team I-deals * TTMS													0.486**	0.146
TCF					0.239	0.120	0.051	0.120						
TTMS * TCF							0.550***	0.144						
*R* ^2^	0.301		0.372		0.397		0.494		0.351		0.342		0.422	
Δ*R*^2^	0.301***		0.071***		0.025***		0.097***		0.351***		0.342***		0.071***	
*F*	6.662***		7.699***		7.515***		9.555***		8.118***		6.867***		8.224***	

*N* = 80. *p < 0.05, **p < 0.01, ***p < 0.001. This table shows the hierarchical regression relationships between each predictor variable and the outcome variable. And “team”-values are calculated by averaging over the team members.

**Secondly, we test hypothesis 2**. Model 1 shows that team I-deals are positively related to team breakthrough innovation (β = 0.355, *p* < 0.001). After adding the mediating variable to Model 3, the result shows that team exploratory-exploitative knowledge sharing is positively associated with team breakthrough innovation (β = 0.294, *p* < 0.01), thus meeting the mediation conditions. In addition, the statistical results of Mplus7.4 shows that the mediating effect value of team exploratory-exploitative knowledge sharing in the relationship between team I-deals and team breakthrough innovation is significant (β = 0.172, 95%CI is [0.090, 0.258], excluding 0). Thus, hypothesis 2 was supported.

**FIGURE 2:**
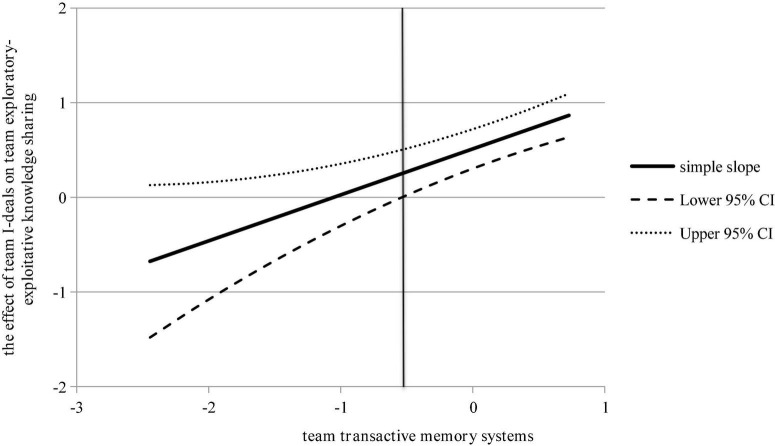
The moderating role of TTMS (J-N). This figure shows the value range of TCF (after decentralization), and what value it takes to show the moderating effect.

**Thirdly, we test hypothesis 3**. Model 7 tested the moderating effect of the TTMS, which was 7.1% better than the overall goodness of fit of model 6, and the interaction term between the team I-deals and TTMS was significant (β = 0.486, p < 0.01), indicating the existence of a moderating effect. Further, we used the Johnson-Neyman (J-N) method to explore the specific form of the moderating effect. The J-N method provides more information by providing confidence bands of simple slopes, which makes up for the inadequacy of the traditional point tracing method to test the moderating effect (M ± 1 SD). As shown in Figure 2, in the part of the TTMS greater than –0.516, the confidence band of the simple slope line excluded 0, so it was significant. The slope line is above 0 axis and slopes to the upper right, indicating that the higher TTMS, the stronger positive association between team I-deals and team exploratory-exploitative knowledge sharing. So hypothesis 3 was supported.

**Fourthly, we test hypothesis 4**. The deviation corrected non-parametric percentile residual bootstrap method was used to test the moderated mediating effect. The result in Table 3 shows that indirect effect of team I-deals (X) → team exploratory-exploitative knowledge sharing (M) → team breakthrough innovation (Y). When TTMS is high (+1 SD), β = 0.221, 95%CI is [0.078, 0.367], 0 is not included, so it is significant. When TTMS is high (–1 SD), β = 0.080, 95%CI is [0.004, 0.190], 0 is not included, so it is significant. At the same time, the difference is significant (β = 0.141, 95%CI is [0.035, 0.307], 0 is not included). Thus, hypothesis 4 was supported.

**TABLE 3:** Analysis results of moderated mediating effect (TMS).

Variable		First stage	Second stage	Direct effect	Indirect effect	Total effect
		X → M	M → Y	X → Y	(P_YM_*P_MX_)	[P_YX_ + (P_YM_*P_MX_)]
		[95% CI]	[95% CI]	[95% CI]	[95% CI]	[95% CI]
TTMS	High	0.752***	0.294**	0.191	0.221**	0.412***
		[0.578, 0.949]	[0.112, 0.451]	[–0.009, 0.381]	[0.078, 0.367]	[0.225, 0.581]
	Low	0.273	0.294**	0.191	0.080	0.272*
		[–0.008, 0.553]	[0.112, 0.451]	[–0.009, 0.381]	[0.004, 0.190]	[0.038, 0.469]
	Difference	0.479**	0	0	0.141*	0.141*
		[0.147, 0.788]	–	–	[0.035, 0.307]	[0.035, 0.307]

*N* = 80. *p < 0.05, **p < 0.01, ***p < 0.001. Bootstrap = 5,000. The table shows that indirect effect of team I-deals → team exploratory-exploitative knowledge sharing → team breakthrough innovation when TTMS at a high or low level.

**FIGURE 3:**
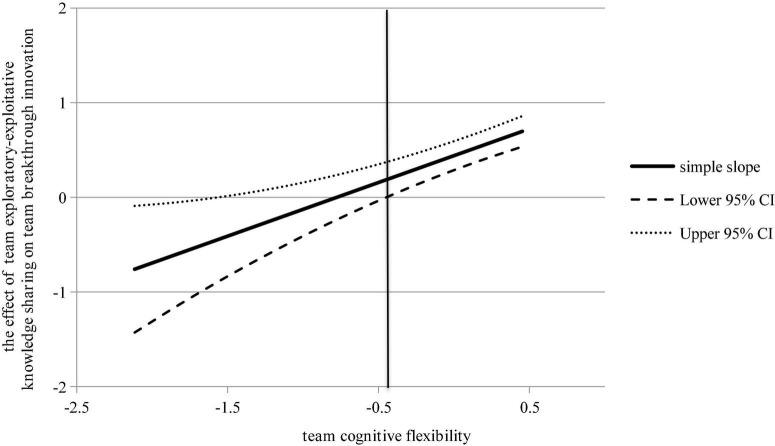
The moderating role of TCF (J-N). This figure shows the value range of TCF (after decentralization), and what value it takes to show the moderating effect.

**Fifthly, we test hypothesis 5**. Model 4 in Table 2 showed that the interaction between TCF and team exploratory-exploitative knowledge sharing were significant (β = 0.550, *p* < 0.001), indicating the existence of moderating effect. Further, the J-N diagram (Figure 3) showed that in the part where TCF was greater than –0.443, the confidence band of the simple slope line excluded 0, so it was significant, and the slope line increases along the *x*-axis, indicating that the higher TCF, the stronger positive association between team exploratory-exploitative knowledge sharing and team breakthrough innovation. So hypothesis 5 was supported.

**Sixthly, we test hypothesis 6**. Table 4 shows that when TCF is high, indirect effect is significant (β = 0.375, 95%CI is [0.214, 0.583], excluding 0). When TCF is low, indirect effect is not significant (β = 0.045 95%CI is [-0.186, 0.193], including 0). At the same time, differences is significant (β = 0.330, 95%CI is [0.072, 0.674], excluding 0). Thus, hypothesis 6 was supported.

**TABLE 4:** Analysis results of moderated mediating effect (TCF).

Variable		First stage	Second stage	Direct effect	Indirect effect	Total effect
		X → M	M → Y	X → Y	P_YM_*P_MX_	P_YX_ + (P_YM_*P_MX_)
		[95% IC]	[95% IC]	[95% IC]	[95% IC]	[95% IC]
TCF	High	0.557***	0.673***	0.105	0.375***	0.480***
		[0.390, 0.717]	[0.405, 1.001]	[–0.116, 0.287]	[0.214, 0.583]	[0.265, 0.644]
	Low	0.557***	0.081	0.105	0.045	0.150
		[0.390, 0.717]	[–0.344, 0.314]	[–0.116, 0.287]	[–0.186, 0.193]	[–0.166, 0.443]
	Difference	0	0.591*	0	0.330*	0.330*
		–	[0.123, 1.157]	–	[0.072, 0.674]	[0.072, 0.674]

*N* = 80. *p < 0.05, **p < 0.01, ***p < 0.001. Bootstrap = 5,000. The table shows that indirect effect of team I-deals → team exploratory-exploitative knowledge sharing → team breakthrough innovation when TCF at a high or low level.

**Seventhly, we test hypothesis 7**. As shown in Table 5, under the conditions of high TTMS and high TCF, the indirect effect of team I-deals on team breakthrough innovation through team exploratory-exploitative knowledge sharing is significant (β = 0.506, 95%CI is [0.072, 0.674]). At the same time, in the other three different combinations, because the 95% CI of indirect effects include 0, they are not significant.

**TABLE 5:** Two-stage moderated mediation effect analysis.

Variable		First stage	Second stage	Direct effect	Indirect effect	Total effect
		X → M	M → Y	X → Y	P_YM_*P_MX_	P_YX_ + (P_YM_*P_MX_)
		[95% IC]	[95% IC]	[95% IC]	[95% IC]	[95% IC]
High TTMS	High TCF	0.752***	0.673***	0.105	0.506***	0.610***
		[0.578, 0.949]	[0.399, 1.005]	[–0.138, 0.285]	[0.268, 0.804]	[0.432, 0.799]
	Low TCF	0.273	0.081	0.105	0.061	0.166
		[–0.008, 0.553]	[–0.360, 0.321]	[–0.138, 0.285]	[–0.285, 0.250]	[–0.243, 0.494]
Low TTMS	High TCF	0.752***	0.673***	0.105	0.184	0.288*
		[0.578, 0.949]	[0.399, 1.005]	[–0.138, 0.285]	[0.031, 0.439]	[–0.008, 0.490]
	Low TCF	0.273	0.081	0.105	0.022	0.127
		[–0.008, 0.553]	[–0.360, 0.321]	[–0.138, 0.285]	[–0.081, 0.132]	[–0.122, 0.355]

*N* = 80. *p < 0.05, **p < 0.01, ***p < 0.001. Bootstrap = 5,000. The table shows that indirect effect of team I-deals → team exploratory-exploitative knowledge sharing → team breakthrough innovation under four combinations of TTMS and TCF at different levels.

**FIGURE 4:**
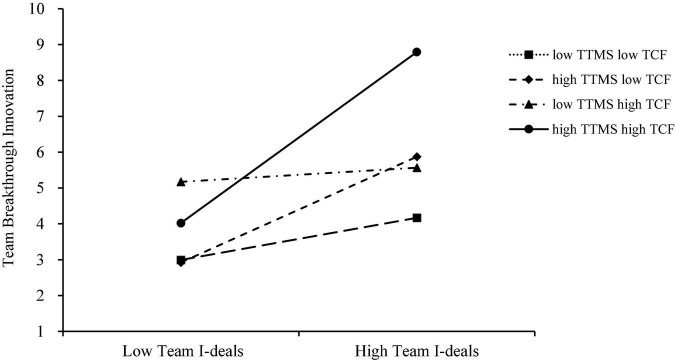
Two-stage combined moderating effect. This figure shows the moderated mediating effect under four combinations of TTMS and TCF at different levels.

Further, this study drew the effect map of joint moderating effect. As shown in Figure 4, under the combined effect of high TTMS and high TCF, the mediating effect of team exploratory-exploitative knowledge sharing in the relationship between team I-deals and team breakthrough innovation is strongest (β = 0.506, *p* < 0.001), thus hypothesis 7 was supported.

## Discussion

### Theoretical implications

Firstly, I-deals and breakthrough innovation have been raised to the team level. In previous studies, breakthrough innovation and I-deals were mostly focused on the individual level, and the discussion on the antecedents of breakthrough innovation was mainly focused on factors such as personal characteristics (Uzunidis et al., 2014), social network relationships of leaders and organizational mechanisms (Gong et al., 2017). The research on the results of I-deals was mainly focused on work engagement (Hornung et al., 2010), OCB (Huo et al., 2014), creativity (Wang et al., 2018), etc. However, this paper extends I-deals and breakthrough innovation from the individual level to the team level, and explores the relationship between team I-deals and team breakthrough innovation. It enriches the research on the antecedents of breakthrough innovation and results of I-deals, which provides a new research perspective for the exploration in the future.

Secondly, it reveals the internal mechanism of I-deals being associated with breakthrough innovation. Existing studies on I-deals are mainly discussed from the theoretical perspectives of social exchange (Liao et al., 2016). This paper is further inclined that the internal mechanism of I-deals may also be a process of team interaction, and uses the I-P-O model to analyze the mediating effect of team exploratory-exploitative knowledge sharing in the relationship between team I-deals and team breakthrough innovation. it responds to the call of research on I-deals from a more diversified theoretical perspective put forward by Liao et al. (2016), and provides new theoretical support for the future research on I-deals.

Thirdly, it widens the moderating effect of relationship between I-deals and innovation performance. A large amount of literature has discussed the main effect and mediating effect, and the exploration of moderating effect is very limited (Abdulsalam et al., 2020; Anand et al., 2021). In order to response to the call of some scholars (Rousseau, 2006), this paper explored and found that the effectiveness of team I-deals will be disturbed by the external environment and team cognitive level. From the perspective of social information processing theory, the moderating effect of its external environment (TTMS) and team cognitive level (TCF) are investigated, which strengthens the effectiveness of team interaction (such as knowledge sharing), and also supports the leadership substitution theory that the team as a unit as a whole, the view that the characteristics of the team (such as external environment and team cognitive level, etc.) can enhance or weaken the effectiveness of incentive measures on the team (Dionne et al., 2002).

### Practical implications

This paper empirically finds that team I-deals are very effective driver to promote team breakthrough innovation, and we hope to provide some practical guidance to organizations implementing I-deals through this paper, mainly including the following three points:

Firstly, organizations need to pay attention to the real needs of valuable teams and adopt team I-deals to stimulate team breakthrough innovation. For example, for teams with special talents (singing, writing, etc.), on the basis of completing their own work, they can arrange tasks that can give full play to their special talents, which is conducive to adding new inspiration for the generation of breakthrough innovation. In addition, we can relax the working place and time appropriately. Teams can choose to work in coffee shops, art exhibitions and other places that are easy to inspire their inspiration, and adopt the result-oriented assessment method for them. In a word, we can give full play to their heterogeneous talents by customizing the work content according to the needs of teams, and promote teams to actively explore how to improve work efficiency and optimize work methods.

Secondly, based on the I-P-O model, the research confirms that exploratory-exploitative knowledge sharing mediates the relationship between team I-deals and team breakthrough innovation. The management enlightenment provided by this result is that organizations should pay attention to the role of team interaction and guide employees to do a good job in knowledge management. The team must need a lot of professional knowledge as support in the process of breakthrough innovation. If properly handled, it can effectively drive employees to participate in breakthrough innovation activities. There are two important issues involved: (1) Pay attention to and identify knowledge sharing; (2) Help the team with knowledge management.

Finally, the study found that TTMS and TCF moderated mediating effect of team exploratory-exploitative knowledge sharing in the relationship between team I-deals and team breakthrough innovation. The results show that for the activities requiring high learning intensity and high uncertainty, such as team breakthrough innovation, on the one hand, the organization should try to establish TTMS through digital technology, strengthen the flexible storage and dynamic management of knowledge; on the other hand, selecting a team with high TCF is more conducive to screening and outputting knowledge and promoting the achievement of the final results. The higher TCF of the team, the more efficient it can make use of the achievements of knowledge sharing, and the more it can identify different types of knowledge, which will amplify the positive impact of team I-deals on team breakthrough innovation in a better TTMS environment. Of course, it is also necessary for leaders to train TCF by creating situations such as fast-paced multitasking (Glass et al., 2013) and using scientific methods (Moore and Malinowski, 2009).

### Limitations and directions for future research

Firstly, although this study collects variable data at different time points, these data are still cross-sectional, so this study can’t make a strict causal conclusion. In future research, we can use the experimental method or collect cross lag panel data to test the causal relationship, so as to provide stronger evidence for the impact effect of team I-deals. Moreover, this study was completed in the China, and its results are bound to be affected by cultural differences (collectivist culture, hierarchical organizational structure, etc.). In future studies, samples from other countries can be considered to ensure the generalizability of the research results. Secondly, this study only explores the impact mechanism of team I-deals from the perspective of I-P-O model. Future research can adopt other theoretical perspectives for broader discussion. For example, according to the affective event theory, as a work event, team I-deals may induce the affective reactions, and then affect the team’s work attitude and behavior. Thirdly, this study does not consider the specific types of I-deals, such as developmental I-deals, flexible I-deals, task I-deals, financial I-deals, etc. (Broschak and Davis-Blake, 2006). Future research can conduct in-depth discussion on different specific types of team I-deals, and build a more systematic and in-depth I-deals mechanism model. Fourthly, this study only discusses the positive effects of I-deals. Previous studies have found that I-deals may also make other organization members jealous, so that the recipients of I-deals may feel threatened and excluded (Ng, 2017). Future research can focus on the dark side of I-deals. Fifthly, in order to fit the research topic of this paper, we choose high-tech enterprises as the research object of this paper. However, I-deals and breakthrough innovation are not limited to high-tech enterprises. In future research, we will also select other types of enterprises as research samples. Finally, this paper selects a younger teams with a long tenure in the company as the research samples, with the purpose of better observing the impact mechanism of I-deals. However, this is also a limitation of this paper. In the future, we will select samples with a wider distribution of age and length of tenure as the research objects.

## Conclusion

Based on I-P-O model, this study discusses the relationship between team I-deals and team breakthrough innovation. The results show that higher team I-deals are indirectly associated with higher team breakthrough innovation through higher team exploratory-exploitative knowledge sharing; TTMS and TCF strengthened the mediating role of team exploratory-exploitative knowledge sharing in the first stage and the second stage respectively; In the case of high TTMS and high TCF, the mediating effect of team exploratory-exploitative knowledge sharing between team I-deals and team breakthrough innovation is stronger.

## Data availability statement

The raw data supporting the conclusions of this article will be made available by the authors, without undue reservation.

## Ethics statement

The study was reviewed and approved by the Academic Ethics Committee of the Hubei University of Technology. Written informed consent was obtained from the individual(s) for the publication of any potentially identifiable images or data included in this article.

## Author contributions

All authors listed have made a substantial, direct, and intellectual contribution to the work, and approved it for publication.
